# Lifestyle Habits and Nutritional Profile of the Spanish Population: A Comparison Between the Period During and After the COVID-19 Pandemic

**DOI:** 10.3390/foods13233962

**Published:** 2024-12-08

**Authors:** Elena Sandri, Lisa Ursula Werner, Vicente Bernalte Martí

**Affiliations:** 1 Faculty of Medicine and Health Sciences, Catholic University of Valencia San Vicente Mártir, c/Quevedo, 2, 46001 Valencia, Spain; 2 Faculty of Teaching and Science of Education, Catholic University of Valencia San Vicente Mártir, c/Quevedo, 2, 46001 Valencia, Spain; lu.werner@ucv.es; 3 Predepartmental Nursing Unit, Faculty of Health Sciences, Jaume I University, Avda. Sos Baynat, s/n, 12071 Castellón de la Plana, Spain; bernalte@uji.es

**Keywords:** healthy lifestyle, diet, survey, Spain, COVID-19

## Abstract

The aim of this study was to investigate the changes in habits regarding the health and lifestyle of the Spanish population during and after the COVID-19 pandemic. A cross-sectional design was used. Data were collected during the pandemic from 22,181 participants and after the pandemic from 3907 participants using the NutSo-HH Scale, assessing demographic, nutritional, and lifestyle variables. Key findings reveal a slight increase in exercise duration (+6.61%) post-pandemic, along with reductions in fried food (−3.57%), juice (−6.45%), and alcohol consumption (−9.66%). Conversely, there were notable increases in the consumption of ultra-processed foods (+2.16%), soft drinks (+6.47%), and coffee/energy drinks (+38.95%). Sleep quality, body image, and self-perceived health showed minor declines. These findings indicate that despite some positive behavioral changes, there is still a significant dependence on unhealthy dietary choices, negatively impacting both mental and physical health. The results highlight the need for public health strategies which encourage healthier eating, increased physical activity, and better sleep quality to reduce the long-term effects of lifestyle changes brought on by the pandemic. Focused interventions are necessary to curb the rising consumption of ultra-processed foods and sugary beverages while fostering overall well-being. This study underscores the vital role of ongoing surveillance and customized public health initiatives to enhance general health in the post-pandemic era.

## 1. Introduction

The health crisis caused by COVID-19 significantly impacted multiple aspects of daily life worldwide, particularly and unprecedentedly altering nutritional habits and lifestyle during both the lockdown periods and the post-pandemic era.

On 11 March 2020 [1], the World Health Organization (WHO) declared the public health emergency caused by the SARS-CoV-2 virus as a global pandemic, and therefore, the majority of European governments [2,3,4,5], including the Spanish government [6], adopted various immediate measures such as lockdowns, mobility restrictions, and social distancing to address the health crisis and control the spread of the disease, which led to unprecedented changes in the way people carried out their daily activities, including those related to health, nutrition, and lifestyle [7]. The effects of the pandemic have not only been physically visible, but they also have had profound psychological, social, and economic consequences, altering health and well-being behaviors in different contexts [8,9,10].

A great impact was noticed on the so-called active lifestyle, as the measures implemented led many people to significantly decrease their physical activities, fostering increased sedentary behavior in the population [11,12]. Additionally, changes were observed in sleeping patterns, with greater difficulty falling asleep and a decrease in sleep quality compared to data from the pre-pandemic era [11,12,13]. Furthermore, nutrition and eating habits were highly affected areas by restrictions, experiencing a major impact both in terms of supply and production [14,15], as well as in the intake of highly processed foods, snacks, and sugary drinks [16,17,18], as unhealthy eating behaviors emerged as a way to cope with isolation, stress, or anxiety [9,13,19,20,21,22].

Studies conducted in various European countries show a widespread increase in the intake of processed products; a reduction in physical exercise; and changes in traditional eating patterns, such as the Mediterranean diet in southern Europe. These well-established eating habits were impacted by economic and availability factors, leading to a reduction in the intake of fresh and more nutritious foods [7,23]. In Spain, the existing literature describes how daily routines and eating patterns were disrupted, with an increase in the consumption of nutritionally poor products [24], alongside a decrease in physical activity [8], particularly during the stricter lockdown periods, all together with alterations in sleeping patterns [13,25,26].

As we can see, there are already numerous studies published worldwide on the effects of the pandemic during the lockdown period; however, few have assessed post-pandemic health behaviors and the long-term effects of the pandemic on the general well-being of the population [27,28,29,30,31,32,33,34,35,36], highlighting key areas where intervention is needed to promote healthier lifestyle and eating habits.

This study aims to address these gaps by analyzing changes in dietary patterns and lifestyle among the adult Spanish population during and after the COVID-19-related restrictions. To achieve this, we utilized the validated Nutritional and Social Healthy Habits (NutSo-HH) scale [37], specifically designed to comprehensively assess both nutritional behaviors and social health-related habits. The application of this tool in the context of evaluating the long-term effects of the pandemic is not only innovative but also provides a more holistic and accurate perspective on the dynamics of lifestyle changes. This novel approach enables the identification of key areas for intervention to promote healthy habits in the post-pandemic era, contributes to mitigating the negative effects of future confinement measures, and enhances the development of evidence-based public health policies.

## 2. Materials and Methods

### 2.1. Study Design and Sampling

A descriptive cross-sectional survey was conducted during two different time periods among adults (aged 18 and older) living in Spain. Participants were excluded from the study if, at the time of completing the survey, they had any medical condition or restriction that could potentially alter their dietary habits, such as hospitalization or confinement.

### 2.2. Ethical Approval

Approval for the study was obtained from the Research Ethics Committee of the Catholic University of Valencia under the approval code UCV/2019-2020/152. Informed consent was obtained from all participants prior to their involvement in the study, and the ethical recommendations contained in the Declaration of Helsinki [38] were followed at all times.

Moreover, before the participants could fill in the questionnaire, they were provided with information about the research which was placed on the initial page of the Google Forms document that hosted the survey. All were asked whether they understood the purpose of the study, whether they agreed to answer the questionnaire voluntarily, and whether they gave their consent for the collected data to be used for the research.

If a participant answered no, the Google Forms page was automatically closed and the participant could not progress in answering the questionnaire.

### 2.3. Instrument

The validated NutSo-HH scale was used for data collection [37] and is divided into several sections: a section on diet that includes the type of diet followed, consumption of various food groups and drinking habits; a section that explores the presence of diagnosed eating disorders or their symptoms; a section on physical activity and health-relevant lifestyle habits; and, finally, a part where anthropometric data such as weight, height, and socio-demographic variables are collected.

### 2.4. Data Collection

Data collection took place during two different time periods. The first occurred during the COVID-19 pandemic in Spain, between August 2020 and November 2021, a time when various preventive measures were still in place. The second data collection period occurred between December 2023 and May 2024, when no more restrictions were implemented. 

The survey was distributed using non-probability snowball sampling [39]. A Google Forms questionnaire was created and shared primarily through online channels, with collaborators helping to disseminate the link. The initial dissemination was carried out via the Instagram account @elretonutricional, as well as through the researchers’ personal social media platforms (WhatsApp, LinkedIn, Facebook, and Twitter) and email distributions to various associations. Additionally, to reach individuals less familiar with digital platforms, physical flyers were distributed in shops and businesses frequented by people from various socio-demographic backgrounds.

### 2.5. Variables

For the nutritional analysis, the IASE index (Índice de Alimentación Saludable para la población Española) was chosen, which was modified to create a condensed version [40]. This adapted index allows a maximum score of 73 points, measuring adherence to the Spanish Society of Community Nutrition (SENC) guidelines [41]. Based on the score obtained, dietary habits are categorized into three levels:

Healthy: IASE score between 58.4 and 73.
Needs changes: IASE score between 36.5 and 58.4.
Unhealthy: IASE score below 36.5.

This classification provides a clear structure to evaluate dietary patterns in the population based on their alignment with national dietary recommendations. A summary table of the variables used in this adapted version of the IASE index can be found in detail in previous articles [42,43].

The other variables related to the frequency of specific food or beverage intake, which have not been used for the IASE index, such as consumption of water, sugary drinks, coffee or energy drinks, and juice and the consumption of fast, fried, or ultra-processed food have been categorized using a 4-point Likert scale, where 1 was assigned to the lowest and 4 to the highest frequency of consumption (for specific details on the score assigned to each of the responses, see Table A1, Appendix A).

The socio-demographic variables considered in the study were categorized as follows:

- Sex: analyzed in a binary manner as male and female.
- Age: divided into four age groups (18–25 years old, 26–40 years old, 41–65 years old, and over 65 years old).
- Level of education: grouped into two categories, basic education (including no formal education, primary or secondary education, vocational training, or baccalaureate) and higher education (bachelor’s, master’s, and PhD).
- Income level: classified as low (household income less than EUR 2200/month), medium–high (household income greater than EUR 2200/month), and no answer.
- Municipality size: based on population size, with three categories established, small municipalities (less than 2000 inhabitants), medium-sized towns (between 2000 and 10,000 inhabitants), and cities (more than 10,000 inhabitants).
- Living arrangements: classified as living alone and not living alone.
- Family life: classified as living with family and living without family.
- Place of residence: categorized based on the various regions of Spain.

Following the same criteria used in previous articles [44,45], variables related to health habits were also categorized using a 4-point Likert scale, with responses ranging from 1 = none or low frequency and 4 = highest frequency. This scale was used to capture the frequency of various health habits such as exercise, smoking, resting, and other lifestyle behaviors. For variables specifically related to eating disorders, a 6-point Likert scale was used to capture a wider range of responses and nuances in behavior (details can be found in Table A1 Appendix A). Finally, body mass index (BMI) and minutes of exercise were treated as numerical variables and not categorized.

### 2.6. Data Analysis

The sample was initially tested for normality using the Shapiro–Wilk test, which revealed that none of the studied variables adhered to normality. This finding was further validated through Q-Q plots, confirming the hypothesis of non-normality [46]. Consequently, ordinal or numeric variables were assessed with the non-parametric Mann–Whitney U test for independent samples. The significance level was set at 0.05. In a second step, we applied a Kendall’s Tau B correlation test to our most important health and behavioral variables to check for correlations between them. 

Additionally, to enhance the interpretation of the statistical significance in a large sample, the effect size r (Rank-biserial correlation coefficient) for the Mann–Whitney U test was incorporated [47]. These measures provided a more nuanced view of the relationships between variables. Specifically, an r value less than 0.3 indicates a small effect [46], while values above this suggest a larger effect [48]. For large samples, effect sizes larger than these thresholds were classified as statistically significant, meaning the relationships between the variables were stronger than what would be expected from random error [47]. All analyses were conducted using Jamovi Version 2.3.28.0.

## 3. Results

Table 1 shows the characteristics of the sample analyzed, contrasting the sample collected during the COVID-19 period with the sample collected afterwards. In the former, N = 22,181 surveys were collected; 4251 were men (19.2%) and 17,930 women (80.8%), with an average age of 34.9 years. In the second set, N = 3907 answers were collected, 585 from men (15%) and from 3322 women (85%), with an average age of 37.1 years.

Regarding almost all socio-demographic factors, as can be seen for example in the educational level, where the majority of both samples (around two thirds) have a higher level of education, the two samples present with very similar characteristics, which undoubtedly facilitates their comparison.

The only exception is in the income level, which presents differences between the two samples: while the sample collected during COVID-19 is fairly uniform (43.9% low income and 47.9% medium–high income), in the post-pandemic sample, the majority of respondents (76.8%) had a low income compared to 16.4% who had an medium–high income.

Table 2 shows a comparison of the nutritional and health variables studied during and after the COVID-19 period. It can be seen that after the COVID-19 period there was a noticeable increase in physical activity (6.61%), from an average of 158.20 min per week to 168.66 min per week, as well as an increase in the consumption of sugary drinks (6.47%) and ultra-processed food (2.16%), while sedentary behavior (−4.46%), sleep quality (−3.24%), smoking (−4.06%), alcohol consumption (−9.66%), and juice consumption (−6.45%) have decreased, and perhaps somewhat surprisingly, the frequency of going out at night has also decreased (−6.72%), but all these differences, as seen by the effect size, are not statistically significant and can therefore be interpreted as noticeable tendencies which still indicate interesting differences in the samples. With regard to the variables related to possible eating disorders, all have experienced a tendency to decrease in the period after COVID-19. On the other hand, the consumption of coffee and energy drinks experienced a statistically significant increase (38.95%) as seen by an effect size over 0.3.

Table 3 analyzes the correlations between the main health and behavioral variables during the third wave of COVID-19 and after the restrictions were lifted. For most of the variables, there is no clear correlation (red cells) both during and after COVID-19.

Predictably, it is observed that the behavioral variables related to eating disorders (no control, body image, and obesophobia) correlate with each other in a medium–strong way (green cells) in the two data sets.

Finally, a moderate to low correlation (yellow cells) is observed, in a similar way both during and after COVID-19, between BMI and the variables of ED, self-perceived health, and weekly minutes of physical activity and sleep quality. The values range from a maximum of 0.3302 obtained between BMI and obesophobia after COVID-19 to a minimum value of −0.2023 between self-perceived health and no control during COVID-19.

## 4. Discussion

The aim of this research was to examine the relationship between characteristics associated with nutrition, health, and behavioral habits, with a particular focus on those related to eating disorders and self-perceived health among Spanish adults. Previous studies have already confirmed that COVID-19 had a negative impact on physical and mental health as well as on health habits worldwide [14,49].

The findings in Table 1 indicate that the regional distribution of the collected answers during and after the lockdown was not homogeneous. As indicated by the percentages, while it was possible to collect more data in regions like Navarre and Catalonia in the post-COVID-19 era, less answers were obtained in Galicia and the Valencian Community, which indicate a regional shift towards these first communities. These differences may be related to variations in the perception of the importance of the study, increased awareness of public health after the lockdown, or specific outreach campaigns in different regions. The likely impact of geographical and demographic variations inside Spain on the observed changes in health and dietary behavior is a crucial factor that has been studied by the authors previously [50]. Amuzu et al. [51] state that the place of residence and the environmental conditions in which an individual resides can influence their health behaviors, including diet, smoking, and physical activity. On the other hand, Carrillo Álvarez et al. [52] claim that differences among rural and urban residents could be due to differences in the overall food-related culture, the ability and competence to actually acquire, conserve, prepare, and eat a varied diet. Rural areas may have shown a reduced inclination to acquire bad habits during and after the lockdown since they depend more on fresh and local foods and have more limited access to these ultra-processed products or sugary beverages [53]. Regarding demographic variations, young people may have been more prone to engage in bad habits including higher energy and sugary drink consumption because of stressors like academic pressure, the social impact of restrictions, and marketing especially aimed at this age range. In contrast, older people may have changed their behavior towards a more mediterranean diet [13].

To achieve the objective of the study, it was decided to use the scale validated for the Spanish population NutSo-HH because it is a multifactorial instrument that allows detailed information to be collected on a wide range of variables that influence health. This scale provides information on the nutritional status of the population as well as on lifestyle aspects such as physical activity, sedentary lifestyles, and rest habits. It also analyzes psychological variables such as those that influence behaviors related to eating disorders or social habits such as smoking or going out at night. Specifically, the NutSo-HH scale has six first-order factors (Mediterranean foods, healthy and unhealthy foods, meats and dairy products, eating disorders, rest habits, and alcohol consumption) and two second-order factors (NUTRI, which includes the factors healthy and unhealthy foods, and meats and dairy products, and HH, which includes the factors eating disorders and rest habits). All these factors are interrelated and have a direct impact on health and well-being; the analysis of these during the lockdown period and afterwards can provide interesting information on how the pandemic has affected the health of the Spanish population.

Our study showed a slight decrease in the BMI post-pandemic (see Table 2); however, this difference was not statistically significant. Similarly, a Lithuanian research study found no changes in BMI among women aged 26 or older [54], as people tended to eat less excessively. In contrast, a study conducted on the Bangladeshi population [49] observed an increase in the prevalence of overweight among both women and men during the COVID-19 pandemic.

Another relevant finding to highlight is that, following the COVID-19 lockdown, the Spanish population noticeably increased the weekly minutes dedicated to physical exercise, as can be seen in Table 2. This result is interesting because, following the lockdown, a global campaign promoting healthy eating and physical activity would have otherwise been crucial in motivating people to return to a healthy lifestyle, while reducing cardiovascular risk [18,55,56,57]. Lockdown, with its negative psychological repercussions, led to unhealthy behaviors such as physical inactivity and increased sedentarism [24,25], even if the more flexible management of free time involved in smart working and home office allowed people to exercise more frequently in the morning [58]. The data from the post-COVID-19 era indicate a positive change in this unhealthy mid-COVID-19 behavior.

From a psychological and social perspective, we can also analyze the observed changes in sedentary behaviors, sleep quality, and nightlife in the population (Table 2), comparing the restriction period and upon returning to the “new normal”. On the one hand, as already stated, the uncertainty and traumatic experiences during lockdown periods led to mood changes, such as anxiety, stress, depression, or boredom, which were associated with reduced physical activity [59] and unhealthy dietary practices [18], prompting people to consume sugary foods and alcohol as coping mechanisms [60,61]. Additionally, lockdown significantly disrupted the circadian rhythms of many individuals, as new habits were established, including irregular sleep–wake schedules, increased screen time, and a more sedentary lifestyle [62,63,64,65]. As a consequence, upon returning to a new normal, many people found it difficult to readjust their natural rhythms, resulting in trouble falling asleep and reduced sleep quality [66,67].

Regarding nutritional habits, the results show that the consumption of unhealthy food, sugary drinks, energy drinks, and coffee did not decrease after the period of restrictions; in fact, their consumption increased (Table 2). These findings are similar to those found in Cyprus [27], China [35], Brazil [36], Latin America [68], Poland [69], Lithuania [70], Germany [71], France [72], Norway [73], and the United Kingdom [74]. Causes attributed to these unhealthy habits include increased stress levels and a decline in well-being [75], along with the tendency to choose food based on mood [76]. Although we see these similar patterns, it is important to consider the cultural and food policy differences between these contexts and Spain. For example, in the United Kingdom, the implementation of a sugar tax (soft drinks industry levy—SDIL) has shown positive results in reducing the consumption of sugary drinks [77]. Spain could also benefit from greater investment in post-pandemic nutritional education strategies, such as campaigns aimed at reducing the consumption of sugary drinks, especially among young people and vulnerable populations. Meanwhile, the consumption of fried foods, tobacco, water, and alcohol decreased in the post-lockdown period, indicating that these products were consumed more frequently during lockdown. Similar results are observed in studies conducted in Poland [78], Denmark [79], Belgium [80], and Bahrain [81]. However, it is important to take into account the contextual differences that may influence these trends. For example, changes in alcohol and tobacco consumption could be influenced by price regulation and public health campaigns, which vary between countries [82].

Compared to the post-lockdown period, lockdown was associated with higher levels of body image dissatisfaction, fear of gaining weight, and lack of control over food quantity consumed. These findings align with a study on the physical, mental, and psychosocial aspects that reported significant negative changes in mental well-being and psychosocial health in the Western Australian community [10]. Another study from the USA concludes that population-based interventions on mental health improvement are recommended given the high prevalence of depression and anxiety [83] and a study conducted in 12 countries of Latin America and the Caribbean reports on the impact of cognitive, emotional, and behavioral variables on subjective well-being [84]. Several European studies have also evaluated these emotional eating disorders associated with the COVID-19 pandemic [85,86], identifying psychological distress as the main cause [87].

As can be seen in Table 3, in our study, a positive correlation was observed between sleep quality and self-perceived health, both during lockdown (in the third wave restriction period) and post-lockdown, highlighting the importance of sleep in health perception and overall well-being. In the pandemic context, changes in routines affected sleep quality for many people, leading to insomnia and disrupted sleep cycles, which increased stress levels and negatively impacted general well-being perception [26]. In the post-pandemic context, it is recommended that sleep habits should be improved by promoting sleep hygiene practices, such as reducing the use of electronic devices before bed and maintaining a consistent sleep routine, as these are crucial for enhancing health perception and reducing stress in affected populations [88].

The positive correlation between physical activity and self-perceived health status (Table 3), measured in minutes, indicates that physical exercise is a key predictor of positive health perception [89], with its impact manifesting differently in periods of lockdown or restriction [24]. During the pandemic, many people experienced a reduction in physical activity due to the lockdown or restrictions, which negatively affected their overall well-being [90,91]. Physical activity also appears to act as a protective factor against stress, especially in the pandemic context, and is considered essential for maintaining a positive perception of health and mental well-being [49].

On the other hand, and continuing with self-perceived health status, we found that it is negatively related to the lack of control over food quantity consumed, indicating that people with a less favorable perception of health tend to struggle with food intake control. In our study, this correlation was found to be significant in the pandemic context due to imposed limitations and routine changes that led to compulsive eating patterns, where food consumption served as a response to stress or negative emotions—a phenomenon commonly observed during the pandemic [70,78].

The positive correlation between BMI and feelings of lack of control over food intake, body image, and obesophobia (Table 3) suggests that individuals who are overweight or obese may struggle to moderate food intake or tend to experience greater body dissatisfaction and fear of weight gain [85,87]. All of these emotional eating disorders associated with COVID-19 have been exacerbated by stress, anxiety, and sedentary lifestyles—factors that have increased the risk of overeating and choosing unhealthy foods, contributing to a perceived loss of control and unhealthy behavioral patterns, such as the avoidance of social activities [92,93].

The positive correlations among the three eating disorder-related factors studied (obesophobia, lack of control, and body image) indicate a significant psychological connection. This suggests that individuals’ relationships with body image and control over eating habits were impacted both during and after the pandemic [94,95]. Research exploring the differences in eating behaviors and body image as confinement measures were lifted will be essential for understanding the full psychological impact of this pandemic and for improving future public health and service planning [96].

Finally, we can conduct a more detailed examination of studies specifically related to the Spanish population to better frame the research within existing national studies. In the study by Rodriguez-Perez et al. [24], the authors emphasize that adherence to the Mediterranean diet increased significantly during the lockdown and an improvement in dietary behaviors was observed. These conclusions are in line with other studies from Italy [97], Croatia [98], Romania [99], Mexico [100], Peru [101], and India [102]. On the contrary, in the studies by Casas et al. [13] and Sanchez-Sanchez et al. [25], it is concluded that even though the adherence to the Mediterranean diet slightly increased during lockdown, the consumption of “unhealthy” food also increased, a statement that was confirmed by authors from France [103], the United Kingdom [104], the USA [105], Brazil [106], and Colombia [107].

### Strengths and Limitations

Among the research’s limitations is that obtaining an identical sample size as in the first survey for the second one was not feasible. This could potentially be attributed to the fact that during the third state of alarm, which occurred between August 2020 and November 2021, people typically had more free time and were more inclined to participate in this type of research. These big differences (N = 22,181 for the COVID-19 period vs. N = 3.907 for the post-COVID-19 period) could limit the liability of the conclusions taken in the discussion. For that reason, the effect size played an important role in the interpretation because it is not as influenced by the sample size as the *p*-value and therefore provides a better measurement tool. On the other hand, N = 3907 is still a noticeable sample size and therefore, taking into account the mathematical law of big numbers, the calculated values for mean and standard deviation should approximate the ones found in the population itself quite well and, in conclusion, give our comparison the needed credibility.

Another limitation of this study is related to the sampling method, which primarily utilized snowball sampling and relied heavily on social media for dissemination. While snowball sampling allows for a broader range of responses, it carries the risk of self-selection bias, as the sample depends on initial participants recommending further participants. On social networks, influencers play a significant role in spreading content related to specific topics, attracting followers with similar interests and behaviors. It is possible that the survey reached some of these communities through influencers, potentially influencing respondents’ behaviors and preferences. Another bias found is related to the age and education of the respondents, with the sample being mostly young and highly educated. This may be due to the dissemination of the questionnaire through social networks where young people have easier access and more familiarity. To minimize those biases, substantial efforts were made to disseminate the questionnaire outside of social networks. In concrete terms, the questionnaire was also distributed physically. Different shops and establishments throughout Spain (chosen for their varied clientele) were contacted and asked to display in their premises a poster explaining the research with a QR code that would send the questionnaire form which allowed customers to participate if they wished to. In addition, an email was also sent to different associations operating in Spain (such as housewives’ associations, scout associations, or charities) asking them to disseminate the survey among their members.

Among the most notable biases in the sample is the gender bias (about 80% of the respondents were female). Despite the authors’ efforts to achieve a balanced sample, this bias could not be avoided, probably because it is structural to this type of research. In fact, it is in line with other studies on the same topic, where a greater predisposition of women to participate in nutrition and health studies has been observed [108,109,110].

In order to try to obtain results that are less influenced by these biases, the authors are currently carrying out a new dissemination of the survey with the specific objective of obtaining a sample with a higher average age and with more people with a basic level of education, and in which the percentage of male participation is higher.

As for the strengths of the study, long-term analyses are essential tools for understanding the impacts the pandemic has had on living patterns over time, particularly regarding dietary and lifestyle changes. Observing how behavioral and health variables evolve over time allows us not only to identify abrupt changes but also to understand if these behaviors remain stable or reverse once the pandemic subsided, thereby enhancing the applicability of the results to other countries and populations, increasing their relevance. This study provides an in-depth analysis of changes in physical activity and their impact on self-perceived health. It thoroughly explores the relationship between physical activity, body mass index (BMI), and perceived well-being, underscoring the importance of these factors on participants’ mental and physical health, particularly within quarantine contexts. This study highlights how psychological factors, such as weight concern and control over eating, influence health perception and lifestyle habits. Including elements such as body image, fear of weight gain, lack of control over food intake, and the impact of stress on eating offers a more holistic understanding of lifestyle changes. This comprehensive perspective adds depth to the findings and enriches the discussion about the effects of the pandemic on the overall health of individuals.

This study not only offers observational data but also provides a broad and well-supported interpretive framework, encompassing the physical, psychological, and social aspects of well-being during times of crisis. Nevertheless, it is essential to continue investigating post-pandemic behaviors to mitigate long-term negative health impacts and strengthen our society’s resilience in facing similar situations in the future. Specific interventions are needed to curb the increasing consumption of ultra-processed foods and sugary drinks while promoting overall well-being.

Future studies should investigate regional and demographic disparities in health behaviors across Spain, emphasizing urban versus rural distinctions and specific cohorts such as younger individuals or those from lower socioeconomic backgrounds. Furthermore, examining the psychological underpinnings underlying habits like stress or emotional eating, together with performing cross-cultural comparisons, may reveal useful public health initiatives. Longitudinal studies are crucial for determining the persistence or evolution of observed patterns, while evaluations of specific interventions, such as educational campaigns or taxes on some products, offer practical insights for permanent enhancements in public health.

## 5. Conclusions

The healthcare crisis triggered by COVID-19 exposed the fragility of our health habits in the face of global emergencies, highlighting the importance of psychological and social factors during this crisis period. We must be prepared to address the negative impact on nutritional and lifestyle habits that the pandemic left behind, including an increased reliance on unhealthy foods that, among other effects, heighten cardiovascular risk. During lockdown, it was crucial to promote healthy eating and physical activity at home, addressing the challenges of sedentary behavior and the use of unhealthy foods as coping strategies for stress and anxiety. Once lockdown was over, a comprehensive reevaluation of the population’s health status was necessary, focusing on both biometric parameters, such as BMI, and psychological factors to enable early identification of persistent anxiety and stress symptoms, thus preventing progression towards potential post-traumatic stress syndrome.

It is essential to implement a global initiative to promote a return to a healthy lifestyle that incorporates a balanced diet and regular physical activity, as these are protective factors that ensure physical and mental well-being, thereby reducing the risk of cardiovascular diseases and other health issues. Such interventions should be holistic, considering both physical and psychological aspects, with the aim of facilitating full recovery and fostering sustainable, healthy lifestyles.

## Figures and Tables

**Table 1:** Characteristics of the sample.

		During COVID-19 Mean (SD) or N (%)	Post-COVID-19 Mean (SD) or N (%)
Sex	Male	4251 (19.2%)	585 (15%)
	Female	17,930 (80.8%)	3322 (85%)
Age in years	Age total (years)	34.9 (11.7)	37.7 (10.8)
	Age male (years)	36.5 (13.4)	36.14 (12.4)
	Age female (years)	34.5 (11.2)	38.0 (10.4)
Age divided by	18–25 years	5952 (26.8%)	484 (12.0%)
categories	26–40 years	9644 (43.5%)	2006 (51.0%)
	41–65 years	6390 (28.8%)	1380 (35.0%)
	>65 years	195 (0.9%)	37 (1.0%)
Exercise in min.	Exercise in min.	158.20 (177.1)	168.66 (179.9)
Education level	Basic education	7027 (31.7%)	1137 (29.1%)
	Higher education	15,154 (68.3%)	2770 (70.9%)
Income level	Low	9727 (43.9%)	3002 (76.8%)
	Medium–high	10,616 (47.9%)	640 (16.4%)
	No answer	1839 (8.3%)	265 (6.78%)
Municipality size	<2000	1014 (4.6%)	186 (4.8%)
	2000–10,000	3587 (16.2%)	734 (18.8%)
	>10,000	17,580 (79.3%)	2987 (76.4%)
Living arrangement	Living alone	2202 (9.9%)	408 (10.4%)
	Not living alone	19,979 (90.1%)	3499 (89.6%)
Family life	Living with family	16,732 (75.4%)	3065 (78.5%)
	Living without family	5449 (24.6%)	842 (21.5%)
Diet	Mediterranean diet	17,573 (79.2%)	2990 (76.5%)
	Vegetarian diet	1425 (6.4%)	220 (5.6%)
	Vegan diet	365 (1.6%)	57 (1.5%)
	Intermittent fasting	1018 (4.6%)	229 (5.9%)
	Raw diet	252 (1.1%)	61 (1.56%)
	Other	1548 (7.0%)	350 (9.0%)
Eating disorder	Diagnosed ED	780 (3.5%)	176 (4.5%)
	No diagnosed ED	21,401 (96.5%)	3731 (95.5%)
Living area	North of Spain	9602 (43.3%)	1772 (45.4%)
	South of Spain	12,579 (56.7%)	2135 (64.6%)
Place of residence	Andalusia	2617 (11.8%)	504 (12.9%)
	Aragon	652 (2.9%)	126 (3.2%)
	Asturias	661 (3.0%)	99 (2.5%)
	Balearic Islands	488 (2.2%)	78 (2.0%)
	Basque Country	925 (4.2%)	175 (4.5%)
	Canary Islands	637 (2.9%)	105 (2.7%)
	Cantabria	217 (1.0%)	37 (1.0%)
	Castilla-La Mancha	729 (3.3%)	136 (4.5%)
	Castile and León	1276 (5.8%)	168 (4.3%)
	Cataluña	3540 (16.0%)	693 (17.7%)
	Ceuta and Melilla	28 (0.1%)	8 (0.2%)
	Community of Madrid	3882 (17.5%)	694 (17.8%)
	Extremadura	395 (1.8%)	56 (1.4%)
	Galicia	1404 (6.3%)	172 (4.4%)
	La Rioja	115 (0.5%)	51 (1.3%)
	Navarre	324 (1.5%)	173 (4.43%)
	Region of Murcia	417 (1.9%)	98 (2.5%)
	Valencian Community	3874 (17.5%)	534 (13.7%)

**Table 2:** Relationship between habit variables in the during COVID-19 and post-COVID-19 groups.

	During COVID-19	Post-COVID-19	Change	*p*-Value * (Effect Size r)
Health values Mean (SD)				
BMI	23.88 (4.03)	23.82 (4.25)	−0.25%	*p* = 0.52 (0.0064)
IASE	53.78 (9.86)	53.95 (9.93)	+0.32%	*p* = 0.11 (0.0156)
Exercise in min.	158.20 (177.1)	168.66 (179.9)	+6.61%	*p* < 0.0001 (0.0429) ^†^
Fried food consumption	2.24 (0.81)	2.16 (0.78)	−3.57%	*p* < 0.0001 (0.0538) ^†^
Fast food consumption	2.37 (0.77)	2.37 (0.76)	+/−0%	*p* = 0.82 (0.0021)
Ultra-processed food consumption	2.32 (0.95)	2.37 (0.97)	+2.16%	*p* = 0.0013 (0.0305)
Water consumption	3.40 (0.64)	3.39 (0.63)	−0.29%	*p* = 0.11 (0.0142)
Soft drink consumption	1.39 (0.67)	1.48 (0.54)	+6.47%	*p* < 0.0001 (0.1282) ^†^
Juice consumption	1.24 (0.53)	1.16 (0.45)	−6.45%	*p* < 0.0001 (0.0639) ^†^
Coffee and energy drink consumption	1.72 (0.71)	2.39 (1.03)	+38.95%	*p* < 0.0001 (0.3743) ^††^
Self-perceived health	3.82 (0.84)	3.80 (0.87)	−0.52%	*p* = 0.22 (0.0112)
Sedentary lifestyle	1.57 (0.83)	1.50 (0.78)	−4.46%	*p* < 0.0001 (0.0468) ^†^
Sleeping hours	2.52 (0.73)	2.48 (0.75)	−1.59%	*p* = 0.0188 (0.0215) ^†^
Getting up rested	2.54 (0.58)	2.54 (0.60)	+/−0%	*p* = 0.7440 (0.0029)
Sleeping quality	3.39 (1.02)	3.28 (1.06)	−3.24%	*p* < 0.0001 (0.0531) ^†^
Smoking	1.23 (0.63)	1.18 (0.55)	−4.06%	*p* < 0.0001 (0.03) ^†^
Alcohol consumption	1.76 (0.87)	1.59 (0.79)	−9.66%	*p* < 0.0001 (0.1015) ^†^
Getting drunk	1.06 (0.29)	1.05 (0.27)	−0.95%	*p* = 0.13 (0.0055)
Nightlife	1.19 (0.43)	1.11 (0.34)	−6.72%	*p* < 0.0001 (0.0661) ^†^
Obesophobia	3.45 (1.44)	3.24 (1.41)	−6.09%	*p* < 0.0001 (0.0836) ^†^
No control	2.78 (1.29)	2.71 (1.25)	−2.52%	*p* = 0.007 (0.0263) ^†^
Body image	3.61 (1.29)	3.44 (1.26)	−4.70%	*p* < 0.0001 (0.0723) ^†^

* Mann–Whitney U test. Comparison between the during COVID-19 and post-COVID-19 groups. ^†^ Tendencies noticeable but not significant, as shown by effect size r < 0.3. ^††^ Significant differences indicated by effect size r > 0.3.

**Table 3:** Correlations between the most important health and behavioral variables in the during and post-COVID-19 samples.

Value	IASE	BMI	Exercise in min.	Sedentary Lifestyle	Sleep Quality	Self-Perceived Health	No Control	Body Image	Obesophobia
During third wave of COVID-19									
IASE	-	−0.041	0.087	−0.0122	0.0556	0.1224	−0.0663	−0.0615	−0.0588
BMI		-	−0.079	0.0193	−0.0632	−0.1530	0.2546	0.1845	0.3076
Sport in min.			-	−0.1123	0.0775	0.2204	−0.0765	−0.0404	−0.0827
Sedentary lifestyle				-	0.0026	−0.0601	0.0579	0.0566	0.0714
Sleep quality					-	0.2609	−0.1478	−0.1408	−0.1343
Self-perceived health						-	−0.2023	−0.1811	−0.1889
No control							-	0.5252	0.6057
Body image								-	0.6628
Obesophobia									-
Post-COVID-19									
IASE	-	−0.0405	0.0698	−0.0007	0.0422	0.1265	−0.0641	−0.0712	−0.0658
BMI		-	−0.0575	0.0053	−0.0577	−0.0872	0.2405	0.2089	0.3302
Sport in min.			-	−0.0889	0.1115	0.2203	−0.1025	−0.0638	−0.0865
Sedentary lifestyle				-	0.0335	−0.0599	0.0284	0.0493	0.0536
Sleep quality					-	0.2502	−0.1706	−0.1630	−0.1380
Self-perceived health						-	−0.1924	−0.1814	−0.1671
No control							-	0.5074	0.5881
Body image								-	0.6533
Obesophobia									-

Kendall’s Tau B. NOTE: Red = No correlation; Yellow = Low correlation; Green = Medium-high correlation.

## Data Availability

The data presented in this study are available upon reasonable request to the corresponding author.

## Appendix A

**Table A1:** Categorization of the health and lifestyle variables and of the variables of eating disorders.

Variable	Category	Score
Sleeping hours	Less than 6 h	1
	Between 6 and 7 h	2
	Between 7 and 8 h	3
	More than 8 h	4
Getting up rested	Never	1
	Very seldom and sometimes	2
	Frequently and almost always	3
	Always	4
Sleep quality	0 and 1	1
	2	2
	3	3
	4 and 5	4
Water	Never, very rarely (2 max. per month), 1 glass/cup/week, and 2 or more glasses/cups/week	1
	2 glasses/cups or less every day	2
	3 to 5 glasses every day	3
	More than 5 glasses every day	4
Sugary soft drinks, coffee, and energy drinks	Never and very rarely (2 glasses max. per month)	4
	One glass per week, and two or more glasses per week	3
	2 glasses or less every day	2
	3 to 5 glasses and more than 5 glasses every day	1
Juice	Never and very rarely (2 glasses max. per month)	1
	One glass per week, and two or more glasses per week	2
	2 glasses or less every day	3
	3 to 5 glasses and more than 5 glasses every day	4
Fish consumption	Never or very seldom	1
	Between 1 and 2 times a week	2
	Three or more times a week	3
	Every day	4
Consumption of fast food, fried, and ultra-processed dishes	Never	1
	Very seldom (2 times a month maximum)	2
	Once a week	3
	Several times a week	4
Getting drunk	Never or less than once a month	1
	Monthly	2
	Weekly	3
	Daily or almost daily	4
Alcohol consumption	Never or once a month	1
	2–4 times a month	2
	2–3 times a week	3
	4–5 times a week or every day	4
Smoking	Non-smoker	1
	Light smoker (less than 5 cigarettes per day)	2
	Moderate smoker (6–15 cigarettes per day)	3
	Severe smoker (more than 16 cigarettes per day)	4
Night outings	Never and sporadically	1
	Between 1 and 2 times a week	2
	More than 3 times a week	3
	Every day	4
Sedentary lifestyle	Less than 7 h	1
	Between 7 and 9 h	2
	Between 9 and 11 h	3
	More than 11 h	4
Obesophobia, no control, and body image	Never	1
	Rarely	2
	Occasionally	3
	Frequently	4
	Very frequently	5
	Always	6
